# Performance Evaluation of Plastic Concrete Modified with E-Waste Plastic as a Partial Replacement of Coarse Aggregate

**DOI:** 10.3390/ma15010175

**Published:** 2021-12-27

**Authors:** Farhan Ahmad, Arshad Jamal, Khwaja Mateen Mazher, Waleed Umer, Mudassir Iqbal

**Affiliations:** 1 Civil Engineering Department, Faculty of Engineering Sciences, National University of Modern Languages, Rawalpindi 44000, Pakistan; engrfarhan51@gmail.com; 2 Department of Civil and Environmental Engineering, College of Design and Built Environment, King Fahd University of Petroleum & Minerals, Dhahran 31261, Saudi Arabia; arshad.jamal@kfupm.edu.sa; 3 Interdisciplinary Research Center of Smart Mobility and Logistics (IRC-SML), King Fahd University of Petroleum and Minerals, Dhahran 31261, Saudi Arabia; 4 Department of Construction Engineering and Management, College of Design and Built Environment, King Fahd University of Petroleum & Minerals, Dhahran 31261, Saudi Arabia; waleed.umer@kfupm.edu.sa; 5 State Key Laboratory of Ocean Engineering, Shanghai Key Laboratory for Digital Maintenance of Buildings and Infrastructure, School of Naval Architecture, Ocean & Civil Engineering, Shanghai Jiao Tong University, Shanghai 200240, China; mudassiriqbal29@sjtu.edu.cn; 6 Department of Civil Engineering, University of Engineering and Technology, Peshawar 25120, Pakistan

**Keywords:** electronic waste, plastic coarse aggregate, green concrete, mechanical properties, durability properties

## Abstract

Plastic electronic waste (E-waste) is constantly growing around the world owing to the rapid increase in industrialization, urbanization, and population. The current annual production rate of E-waste is 3–4% in the world and is expected to increase to 55 million tons per year by 2025. To reduce the detrimental impact on the environment and save natural resources, one of the best solutions is to incorporate waste plastic in the construction industry to produce green concrete. This study examines the use of manufactured plastic coarse aggregate (PCA) obtained from E-waste as a partial replacement of natural coarse aggregate (NCA) in concrete. Six types of concrete mix with 10%, 20%, 30%, 40%, and 50% substitution of NCA (by volume) with PCA are prepared and tested. This study investigates the effect of manufactured PCA on the fresh and hardened characteristics of concrete. The properties of recycled plastic aggregate concrete (RPAC) studied include workability, fresh density, dry density, compressive strength (CS), splitting tensile strength (STS), flexural strength (FS), sorptivity coefficient, abrasion resistance, ultrasonic pulse velocity (UPV), and alternate wetting and drying (W–D). The results indicate that the CS, STS, and FS of RPAC declined in the range of 9.9–52.7%, 7.8–47.5%, and 11–39.4%, respectively, for substitution ratios of 10–50%. However, the results also indicate that the incorporation of PCA (10–50%) improved the workability and durability characteristics of concrete. A significant decrement in the sorptivity coefficient, abrasion loss, and UPV value was observed with an increasing amount of PCA. Furthermore, RPAC containing different percentages of PCA revealed better results against alternate W–D cycles with respect to ordinary concrete.

## 1. Introduction

Concrete is the most broadly used construction material in the building industry [1]. It is an extensively used man-made construction material and is often said to be the second most essential substance consumed on Earth, after water [2,3]. The rapidly growing industrialization, urbanization and population significantly increased the demands and developments in the construction industry [4]. Concrete has gained a vital role in the construction industry because of its low price, the convenience of its raw materials, high compressive strength and durability [5]. The properties of concrete are significantly influenced by the characteristics of aggregates which typically occupy a total of 65–80% of its volume [6]. It is predicted by the global aggregates construction market that aggregate demand will be increased up to 59% by the end of 2025 [7]. Nowadays, many countries, due to a shortage of natural resources, are facing problems and depend on imports to fulfill their demands [8]. Concrete ingredients are used on a large scale, and the usage of coarse aggregates from rocks causes a particularly fast reduction in natural resources, thus welcoming disasters in the form of global warming and land sliding. To reduce the detrimental impacts associated with concrete production [9,10], the conservation of raw materials needs to be emphasized. Coarse aggregates typically occupy 65–70% of the concrete’s total volume. Efforts are required by the modern world to take important steps in order to save nature without compromising on the overall performance of concrete. In the past, several attempts have been conducted with the aim of replacing natural aggregate with recycled aggregate [11,12,13,14,15]. Therefore, several other alternatives in concrete to natural aggregates such as demolition waste, plastic waste, paper, etc. have gained momentum. The use of recycled solid waste aggregates [16,17], plastic [18], glass [19], scrap tires [20], cardboard [21], electronic waste [22], etc., in concrete are also investigated in several studies.

Nowadays, the use of modern electronic appliances turns out to be an important part of our daily life. Due to the technological innovation, upgrade and advancement of technological products, the rate of obsolescence of electronic equipment has also increased, thus making E-waste an emerging waste in the world. Compared to other waste products, the degradation process of E-waste is comparatively more challenging, thereby significantly damaging the environment. It was reported that in 2013, the production of plastic products reached around 299 million tons [23]. The Environmental Protection Agency (EPA) of the United States (US) estimates that annually about 6–10% of E-waste generation increases, out of which only 5% is recycled globally [24]. Hence, the appropriate recycling and disposal of E-waste products are needed to protect the environment from its hazardous, adverse, and detrimental effects. The best solution to challenge this problem is to incorporate waste plastic in the construction industry. The reuse of E-waste as a substitute for aggregate in concrete production can help in mitigating and addressing the environmental pollution problems related to plastic. Recycling E-waste is an effective technique for minimizing solid waste, reducing the hazardous and harmful environmental impacts [12]. With respect to natural aggregate, the E-waste aggregate is lighter in weight, and thus it can minimize fuel consumption during conveyance and its associated costs. Additionally, its production cost is relatively less. Therefore, plastic concrete can be used as a lightweight material that is correlated with various advantages, i.e., ease in handling during consumption, reducing the efforts in manufacturing, and providing adequate thermal insulation [25]. In addition, earthquake forces depend on the structure’s self-weight and the use of plastic aggregate can decrease the self-weight of concrete structures, which can minimize the effect of earthquakes [26].

While investigating the strength properties of E-waste plastic concrete, Needhidasan et al. [27] studied the effect of shredded E-waste on concrete performance. The replacement ratios of plastic waste ranged from 0 to 20% by volume of coarse aggregate. It was examined that the decline in compressive strength (CS) and flexural strength (FS) occurs with the rise of E-waste, but the tensile strength increased. Additionally, the usage of grinded E-waste in concrete production is an environmentally friendly solution and reduces the cost and unit weight of concrete. Sabau et al. [28] examined the decrement in CS when the coarse aggregates were partially substituted by different proportions of E-waste, i.e., 40%, 50%, and 60%. Compared to the control mix, a maximum decrease in CS and density of 44% and 22%, respectively, was observed. Tafheem et al. [29] reported the influence of plastic waste such as high-density polyethylene (HDPE) and polyethylene terephthalate (PET) on the performance of concrete. The replacement ratios of plastic waste ranged from 0 to 10% of coarse aggregate (by volume). The results describe that at 10% substitution of coarse aggregate by PET aggregate, the minimum decrement in CS was 35%, whereas in splitting tensile strength (STS), a 21% increase was observed. Additionally, a 4% reduction in fresh unit density was reported. Kumar et al. [30] found that when coarse aggregate is partially substituted by plastic aggregate up to 50% with an increment of 10%, the mechanical properties compared with the control concrete were remarkably reduced. Rathore et al. [31] investigated the influence of different percentages of E-waste plastic, i.e., 5%, 10%, 15%, 20%, 25%, and 30% on the behavior of concrete and found that the CS and FS of concrete containing 15% E-waste aggregate were, respectively, reduced by 20.35% and 15.69% with reference to the control mix. They reported that for a construction purpose greater than 15%, the substitution of E-waste is not satisfactory. Zeeshan et al. [32] performed a comprehensive experimental analysis by using plastic aggregate as a partial substitution of coarse aggregate. The replacement ratios of plastic waste ranged from 0 to 20% of coarse aggregate. It was revealed that the mechanical characteristics were reduced by substituting 10 and 20% of coarse aggregate by E-waste, whereas an enhancement in workability and durability characteristics was reported.

Several researchers investigated the effect of different additives in E-waste incorporated concrete. Prasanna et al. [14] investigated the influence of fly ash on the performance of concrete containing grinded E-waste aggregates. The replacement ratios of E-waste aggregates ranged from 0 to 20%, with an increase of 5%. It was reported that the CS declined by 33.7% when 20% of grinded E-waste was incorporated into concrete, which indicates that substitution beyond 20% is not satisfactory. The results also reveal that when 10% fly ash was introduced in plastic concrete containing 20% E-waste, a decrement in CS was observed from 33.7% to 16.86%. Nadhim et al. [33] observed the influence of fly ash on the performance of concrete containing E-waste. Akram et al. [34] studied the influence of E-waste aggregates on the concrete performance in which NCA has been partially replaced with shredded plastic aggregates. The results report that a 34% decline in CS was observed when 15% of E-waste aggregates were incorporated, and it was also observed that the results resemble those of the control mix when 10% of fly ash was introduced.

Alongside the strength characteristics, durability is an important aspect of structural concrete. To examine the durability characteristics of plastic concrete, durability tests were also carried out [35,36], which reported that concrete with plastic aggregate is acceptable and has better results with respect to control concrete. It was revealed that the plastic concrete seems to be satisfactory for utilization in aquatic structures as the durability characteristics are of major concern in marine structures [5], and compared with control concrete, the plastic concrete has shown higher resistivity against sulfate and chloride attack [8,37]. Additionally, from previous studies, it was reported that the workability of concrete containing plastic aggregates increases with respect to control concrete, owing to the zero water absorption capacity of plastic aggregate [11,15]. The abrasion resistance of plastic aggregates was rarely investigated and reported as greater than natural aggregates because of their improved toughness [38]. Zeeshan et al. [32] found that the ultrasonic pulse velocity (UPV) value declines with increasing proportions of plastic aggregate in concrete. It was observed that the UPV value decreased by 1.2%, 1.9%, and 3.3% at 10%, 15% and 20% partial substitution of NCA with PCA, respectively. However, the behavior of manufactured plastic aggregate concrete when exposed to alternate wetting and drying (W–D) has not been examined to date.

A brief assessment of the current literature indicates that the several types of research carried out on plastic concrete have focused on aspects of non-manufactured E-waste aggregates such as cleaning, sorting, and grinding or shredding the E-waste. The literature review suggests that a comprehensive study associated with the behavior of recycled plastic aggregate concrete (RPAC) containing manufactured plastic coarse aggregate (PCA) is missing. This study focused on utilizing the manufactured PCA through the proper heating procedure [32] of shape and size comparable to NCA. To introduce plastic concrete as a practical alternative, it is crucial to investigate the several important characteristics of this new concrete type. This study intended to explore the fresh properties (i.e., workability and fresh density) and hardened properties in terms of mechanical properties (dry density, CS, STS, and FS), and durability properties (abrasion resistance, alternate wetting and drying, sorptivity coefficient, and UPV) of concrete by incorporating different percentages of PCA (10–50%) by volume of NCA.

## 2. Materials and Methods

### 2.1. Materials

Ordinary tap water is used in concrete mixes for hydration purposes, having a pH value range between 6.5 and 7, as per ASTM C1602/C1602M-12 standard [39]. Ordinary Portland cement (OPC) Type-I, as per ASTMC150 [40] standard, was used as a binder. Table 1 shows the physical and chemical properties of cement. The oxide composition of cement was obtained experimentally through the X-ray fluorescence (XRF) technique (JEOL, Tokyo, JAPAN). The physical properties of cement such as consistency and setting time were determined using Vicat’s apparatus (Gilson Company, Lewis Center, OH, USA), whereas fineness modulus, soundness, compressive strength, and specific gravity were determined using the particle size distribution method, Le-Chatelier method, 50 mm cube strength, and Le-Chatelier’s flask method, respectively. Locally available “Lawrencepur sand” was utilized as fine aggregate, having a fineness modulus of 2.27, and the maximum size of particle was 4.72 mm as per ASTM C-33 standard. The coarse aggregate used in this research work had minimum and maximum particle sizes of 4.75 mm and 20 mm, respectively, and was obtained from Taxila (Margalla brand) (Stone Crush Supply Company, Taxila, Pakistan), Pakistan. The general characteristics of the aggregates are summarized in Table 2.

### 2.2. Manufacturing Procedure of E-Waste PCA

**Figure 1:**
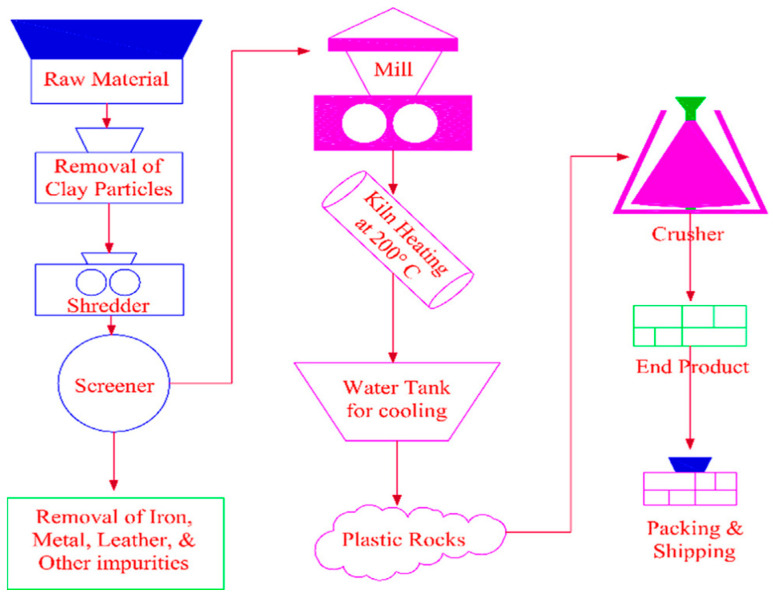
The manufacturing procedure of PCA [2].

**Figure 2:**
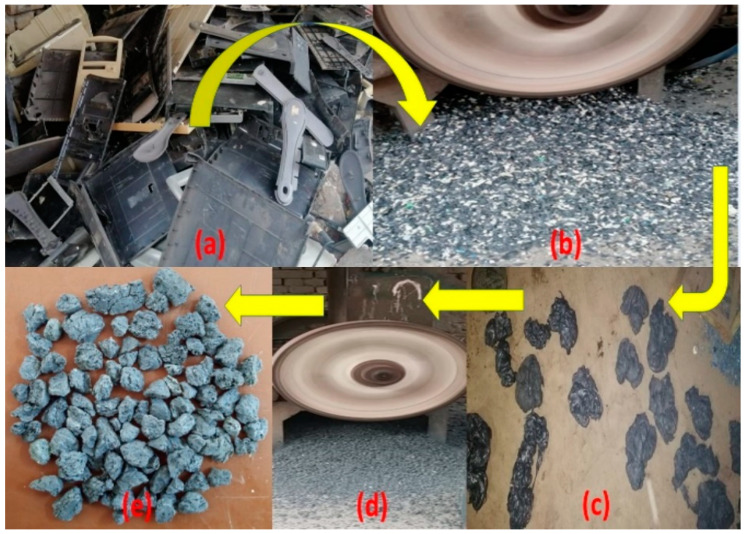
Plastic aggregate manufactured: (**a**) Raw E-waste; (**b**) Crushed raw E-waste; (**c**) Plastic rocks; (**d**) Electric crusher; (**e**) PCA [4].

**Figure 3:**
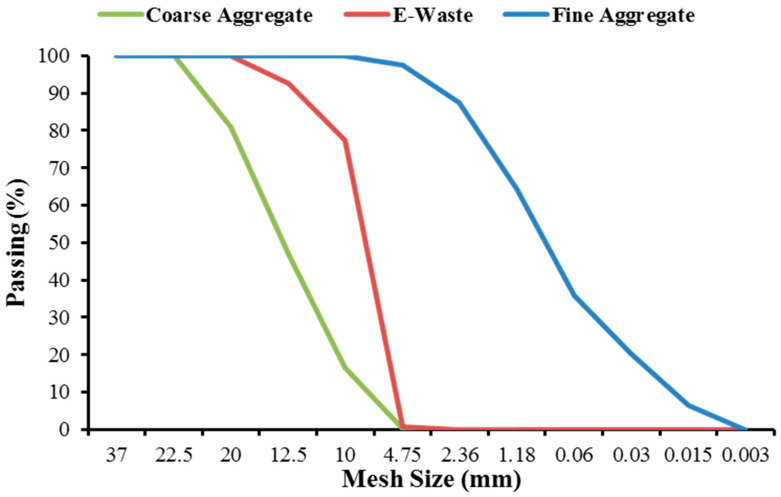
Granulometry analysis of sand, NCA, and E-waste PCA.

The E-waste utilized in this research work belongs to the family of acrylonitrile butadiene styrene (ABS) plastic, which consists of polycarbonate and acrylonitrile butadiene styrene. In previous works, ABS plastic was studied and its composition and other features were documented [41]. The plastic used for manufacturing artificial aggregate was obtained from E-waste, i.e., it was derived from the casing of different electronics equipment. Scrap/waste electronic equipment, keyboards, TVs, computers, mouse, LCDs, monitors, etc., created the E-waste plastic. These plastics are non-biodegradable and were procured locally and changed to the appropriate size and shape of plastic coarse aggregate (PCA), comparable to natural coarse aggregate (NCA) through a suitable manufacturing procedure. Figure 1 displays the schematic illustration of the whole manufacturing technique. The plastic aggregates are manufactured in four different stages from raw E-waste. Firstly, by using tap water, the raw plastic E-waste (Figure 2a) is washed, cleaned, and dried. Secondly, raw E-waste is crushed into small flakes or shredded particles through an electric crusher, and grinded E-waste particles were screened out to eradicate objects other than plastic such as steel, wires, leather, etc., as shown in Figure 2b. In the third step, shredded particles of waste plastic were heated at 200 °C in a kiln and the shredded particles were melted. After melting, plastic rocks were obtained on cooling as indicated in Figure 2c. Finally, the plastic rocks were crushed to obtain PCA of size and shape equivalent to NCA, as given in Figure 2d,e. Table 2 displays the general properties of manufactured PCA. Figure 3 indicates the granulometry analysis of sand, NCA and PCA.

### 2.3. Concrete Mix Proportions

M25-grade concrete having a mix ratio of 1:2.14:3.08 (cement: fine aggregate: coarse aggregate) was prepared in this work [30]. The maximum size of fine and coarse aggregate was 4.72 mm and 20 mm, respectively, and the water cement ratio was kept at 0.49. Six concrete mixes were prepared with one control mix and five mixes of RPAC, including 10%, 20%, 30%, 40% and 50% PCA by volume of NCA, respectively. These mixes were named CM, PCA10, PCA20, PCA30, PCA40, and PCA50. The mix names were designated as such because the PCA is plastic coarse aggregate, CM is control mix, and 10, 20, 30, 40, and 50 are the different percentages of PCA. Table 3 shows the details of the concrete mix proportions. The preparation method adopted was in accordance with the previous study [32]. To mix concrete, a tilting drum was used having a revolving speed of 35 rpm (revolutions per minute). The concrete mix was prepared in 2 stages. Initially, the aggregates such as fine aggregate, NCA and PCA were mixed for 4 min along with 75% water. Furthermore, for the next 4 min, cement was also added along with 25% water.

### 2.4. Test Methods

The experimental program of the present study, along with the number of specimens tested and standards used, is listed in Table 4, whereas Table 5 shows the list of equipment details alongside the corresponding manufacturer country. The workability of RPAC was determined using the slump test as per standard ASTM C143 [42]. The fresh density of RPAC was carried out as per standard ASTM C138/C138M [43]. The dry density of RPAC was evaluated in the saturated surface dry (SSD) condition after 28 days by using an analytical balance determining its size and weight. For each test result, 3 samples were tested, and the average result was considered.

The mechanical characteristics of concrete containing plastic aggregate were determined based on CS, STS, and FS tests. To determine the CS and STS, cylindrical samples of standard dimension (150 mm in diameter × 300 mm in height) after a 28-day curing period were tested as per ASTM C39/C39M-12 and ASTM C496/C496M-17 [44,45], respectively. For CS and STS tests, a total of 36 samples were prepared. Samples were cast and finished in a laboratory. Demoulding of samples was carried out after 24 h and kept in water until tested. A universal testing machine (UTM) (Aoki construction equipment, Okayama, Japan) was used for CS and STS tests having a 1000 kN load capacity. The FS test determines the ability of a concrete specimen to counter bending loads and it was carried out as per standard ASTM C78/C78M-18 [46]. A flexural testing machine working under the principle of three-point loading was used to determine the flexural strength of cementitious composites. Eighteen prismatic specimens having dimensions of 100 × 100 × 400 mm^3^ were prepared and tested after a 28-day curing period to determine their bend strength.

The durability properties of RPAC were examined based on the abrasion resistance, alternate wetting and drying (W–D), sorptivity coefficient, and UPV test. The sorptivity coefficient was determined as per ASTM C1585-04 [47] and the dimensions of the tested sample were of 50 mm thickness and 100 mm diameter. The samples were sealed along with their thickness and uppermost top surface, to prevent the ingression of the side water, and only the lower surface of sample was unsealed, which was subjected to water. The ingression of the water in the sample took place through the capillary rise and an increase in the weight of the sample was observed and measured [4]. The coefficient of sorptivity was evaluated using Equation (1).

(1) $$S=\frac{I}{t^{\frac{1}{2}}}$$

where I denotes the water absorption (cumulative) per unit area of the exposed surface, and t represents the time of exposure (in a minute). The parameter S measured in mm/min^1/2^ is the coefficient of sorptivity, and I=ΔW/Ad, $\Delta W=W_{2}-W_{1}\;$where *W*_1_ denotes the dry weight of specimen (in grams), *W*_2_ represents the specimen’s weight after 4 hours ingression of water through the capillary rise, A denotes the surface area of the unsealed surface exposed to water, and d is the water density

The abrasion resistance of RPAC was measured as per ASTM C131/C131-20 standard [48]. Cylindrical specimens of dimension 100 mm × 150 mm were prepared. Los Angeles (LA) apparatus (Turkish Exporter, Istanbol, Turkey) was used to measure the abrasion resistance of specimens. The LA apparatus rotates at the speed of 30 rpm for 300 revolutions without using a steel ball. As a result of the abrasion test, the percentage reduction in mass of the concrete specimen was evaluated using Equation (2).

(2) $$Loss\;in\;Mass\left(\%\right)=\left[\frac{M1-M2}{M1}\right]\times 100$$

where M1 is the specimen mass before the abrasion test and M2 is the specimen mass after the test.

To examine the influence of alternate wetting and drying (W–D) on the CS of plastic concrete, cubic specimens of 100 mm in size were prepared. Samples were subjected to 25 and 50 alternate W–D cycles after a curing period of 28 days. The duration of one complete cycle was 48 h (two days), in which the specimens were kept in water for the first 24 h, and then for the next 24 h, the samples were taken out from the water and then exposed to the air in the lab to dry [32,49]. The specimens were tested after 25 and 50 cycles, and the loss of CS for each specimen was recorded.

To determine the consistency, quality, and uniformity of concrete specimens, the UPV test was carried out [4]. Cylindrical samples of dimension 150 mm in diameter × 300 mm in height were used to evaluate the UPV value as per ASTM C597-09 standard. This test was conducted after a curing period of 28 days. In this test, both sides of the cylindrical specimen were provided with transducers (transmitter and receiver) of the frequency range 55 kHz. The time of travel of the ultrasonic pulse between the two transducers was noted and the UPV value was obtained by dividing the length of the specimen by time of travel.

## 3. Results and Discussion

### 3.1. Workability

**Figure 4:**
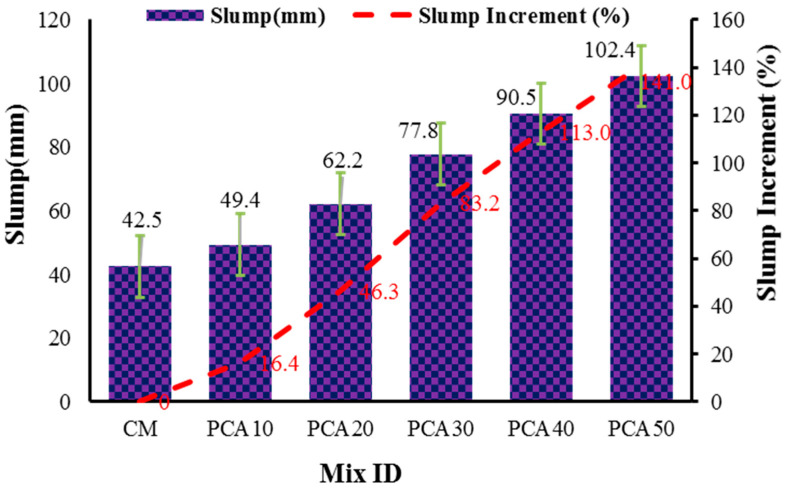
Workability of RPAC.

Workability measures the ability of freshly mixed concrete to flow without segregation. It is a vital property of concrete that ensures proper handling and controls concrete’s strength and durability. The workability of RPAC was examined via slump test and Figure 4 displays the results for different concrete mixes. The results reveal that the workability of RPAC significantly increased with the increase in the substitution ratio of PCA compared to the control mix. The presence of non-absorbent plastic aggregate in concrete increased the workability of RPAC. The zero water absorption capacity of PCA results in excess water in the paste, thereby increasing the workability. Past works also reported a similar finding of an increase in workability [33,50,51]. However, few studies [52,53,54] mentioned a decline in the workability of concrete containing plastic aggregates owing to the irregular size and shape of shredded plastic aggregates. In the present study, manufactured PCA was used with proper control over the size and shape, thus yielding higher workability with respect to mineral aggregate concrete. The detailed procedure for the manufacture of PCA is presented above (Section 2.2). With reference to control mix, the workability of plastic concrete increased by 16.4%, 46.3%, 83.2%,113%, and 140.9%, respectively, at 10%, 20%, 30%, 40%, and 50% replacement of NCA with PCA. The existence of PCA in the range of 0% to 50% increased the slump value from 42.5 mm to 102.4 mm, an increase of 140.9%.

### 3.2. Fresh and Dry Density

**Figure 5:**
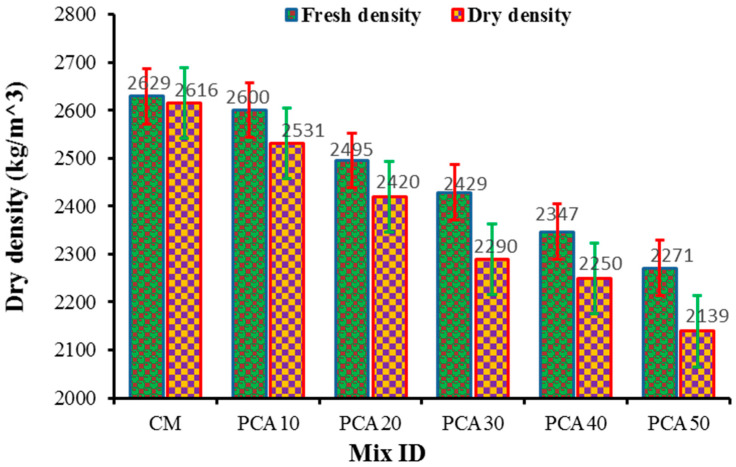
Fresh and dry density results.

The density of concrete depends upon the unit weight of concrete’s ingredients and their mix proportions. Figure 5 displays the fresh and dry density of RPAC containing different percentages of PCA. With reference to the control mix, the fresh and dry density of plastic concrete declined. This decrement in density is owing to the presence of PCA, which has a lower unit weight compared to NCA. The results show that the fresh density of concrete containing PCA as alternative coarse aggregate was 2600 kg/m^3^, 2495 kg/m^3^, 2429.7 kg/m^3^, 2347.7 kg/m^3^, and 2272 kg/m^3^, respectively, at 10%, 20%, 30%, 40%, and 50% substitution level. With respect to the control mix, the maximum and minimum reduction in fresh density was 13.6% and 1.10%, respectively, at 50% and 10% replacement of NCA with PCA. In the literature, there is agreement that the density of concrete containing PCA decreases because of the low specific gravity of plastic aggregates with respect to mineral aggregates. A similar pattern of decrease in fresh density is well reflected in previously published works [30,50]. Reduction in density is directly related to the substitution level of aggregates [51]. In the current study, the maximum reduction in dry density is 18.2% for the PCA50 specimen with reference to the control mix. Even though the PCA decreased the concrete’s density, the values were still well above the utmost limit for lightweight concrete. A comparatively smaller decrement in dry density was observed in the current study with manufactured PCA with respect to grinded plastics in past works, ascribed to the size and shape of PCA, which was similar to NCA.

### 3.3. Mechanical Properties

#### 3.3.1. Compressive Strength

**Figure 6:**
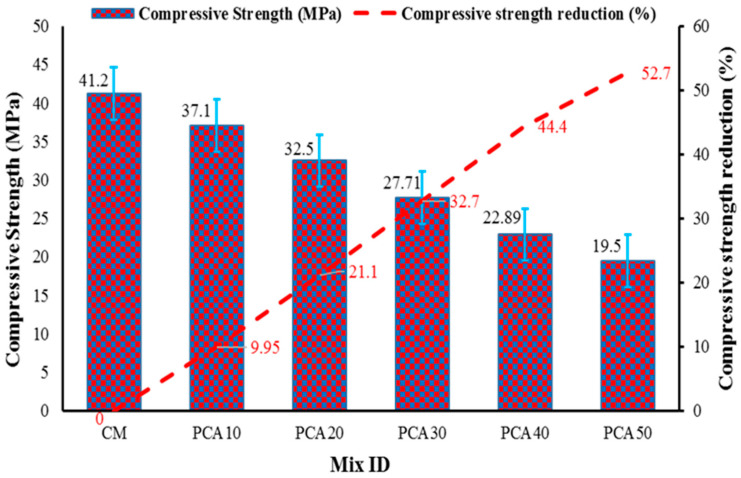
CS results of RPAC at 28 days.

**Figure 7:**
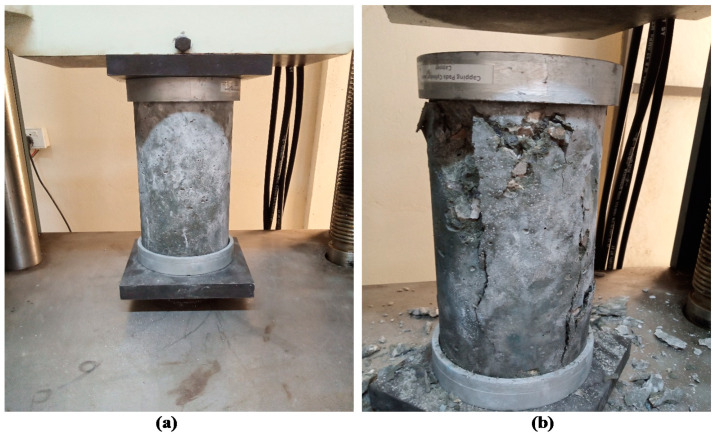
Compression test assembly: (**a**) cylinder before test and (**b**) cylinder after test.

Compressive strength (CS) is an essential property of concrete that measures its ability to carry loads and is considered as one of the essential parameters in reinforced concrete design [52]. The CS of cylindrical specimens containing different proportions of PCA was evaluated as per ASTM C39 [45] standard. The CS of 28 days of cured concrete containing different percentages of PCA is shown in Figure 6. CS of control mix came out to be 41.2 MPa, while concrete with 10%, 20%, 30%, 40%, and 50% PCA revealed compressive strengths of 37.1 MPa, 32.5 MPa, 27.71 MPa, 22.89 MPa, and 19.5 MPa, respectively. The maximum reduction in CS was 52.7%, observed at 50% replacement level, whereas a minimum reduction of 9.95% was observed at 10% replacement level compared to the control mix. The results show that as the substitution level of NCA with PCA increased, the CS of RPAC decreased significantly with respect to the control mix, which is in compliance with the past works [14,34,51]. Figure 7 shows the CS test assembly along with a cylindrical specimen before and after the test. The failure mode of the specimen is indicated in Figure 6b, which shows that rupture failure occurs in the specimen containing PCA, ascribed to the weak bond formation between PCA and concrete ingredients. The plastic aggregates examined after failure revealed that the plastic aggregate does not break because of its flexible nature. Due to the smooth texture of PCA, weak adhesion develops between plastic aggregate and cement paste, thereby resulting in a decrement in strength. Additionally, the strength decrement may be due to the presence of non-absorbent plastic aggregate, which results in excess free water in cementitious composites. In addition, plastic aggregate has less density, unit weight, rigidity, and strength compared to NCA, thus creating a high-stress region and facilitating the spread of damage, which may also be one of the reasons for the decline in strength [53,54]. Therefore, a replacement proportion of manufactured PCA up to 50% can be utilized as the CS obtained is more than 17.24 MPa, which is the lowest compression capacity normally suggested for structural concrete [55].

#### 3.3.2. Splitting Tensile Strength

**Figure 8:**
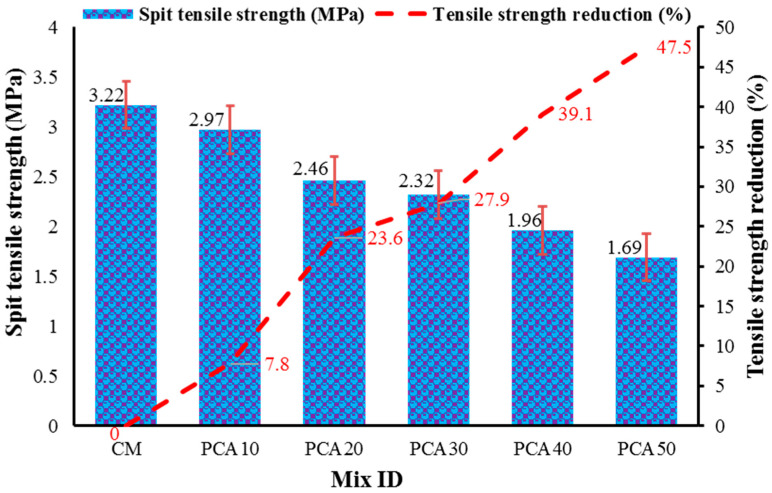
STS results of RPAC at 28 days.

Figure 8 shows the variation in split tensile strength (STS) results for RPAC mixes containing different percentages of PCA. The STS of the control mix came out to be 3.22 MPa at 28 days, while concrete with 10%, 20%, 30%, 40%, and 50% PCA replacement of NCA showed STS of 2.93 MPa, 2.69 MPa, 2.46 MPa, 2.14 MPa, and 1.88 MPa, respectively. The maximum reduction in STS was 47.5%, observed at the 50% replacement level, whereas a minimum reduction of 7.8% was reported at the 10% replacement level, with respect to the control mix. The results show that the STS of RPAC declined significantly as the substitution level of NCA with PCA increased. This decrement in the STS is owing to the weak bond or adhesiveness between PCA and cement paste. Due to the smooth texture of PCA, weak adhesion/bonding develops between plastic aggregate and cement paste, thereby resulting in a decrement in strength [56]. Additionally, PCA has less density, unit weight, rigidity, and strength with respect to NCA, hence producing a high-stress zone that facilitates the spread of damage, which is also considered as the reason for strength reduction [53,54]. The influence of manufactured plastic aggregates on the STS of RPAC is consistent with the findings of past studies [11,56].

#### 3.3.3. Flexural Strength Test

**Figure 9:**
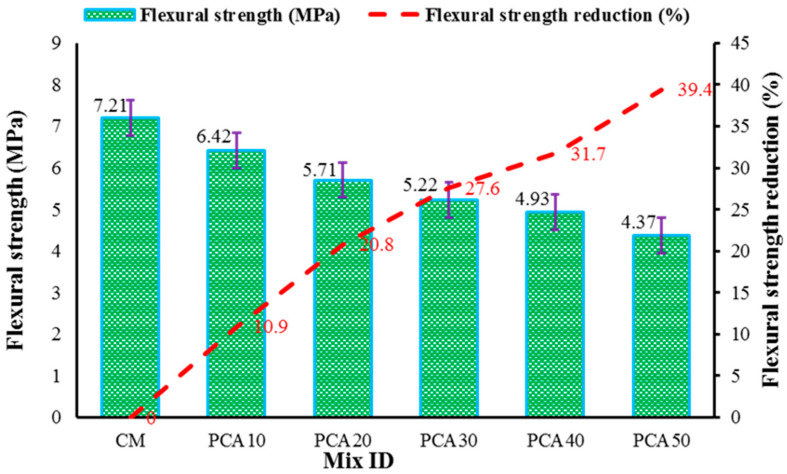
Flexural strength results of RPAC at 28 days.

The flexural strength (FS) test, also known as transverse rupture strength, measures the ability of concrete to counter bending loads and was carried out as per ASTM C78/C78M-18 [46] standard. Figure 9 shows the FS results of RPAC with different replacement levels of NCA with PCA and the effect of PCA on FS. The results show that similar to CS and STS, the FS of RPAC reduced with the increase in the plastic aggregate content. The results show that the FS of concrete containing PCA as alternative coarse aggregate was 7.21 MPa, 6.42 MPa, 5.71 MPa, 5.22 MPa, 4.93 MPa, and 4.37 MPa when the substitution level was 10%, 20%, 30%, 40%, and 50%, respectively. With reference to the control mix, the maximum reduction in FS was 39.4%, observed at the 50% replacement level, whereas a minimum reduction of 10.9% was observed at the 10% replacement level. The reduction in FS of RPAC is ascribed to the smooth surface of the plastic aggregate, thereby yielding a weak bond/adhesiveness between cement paste and PCA. The effect of PCA upon the FS of RPAC is well reflected in previously published works [27,30,36].

### 3.4. Durability Properties

#### 3.4.1. Abrasion Resistance

**Figure 10:**
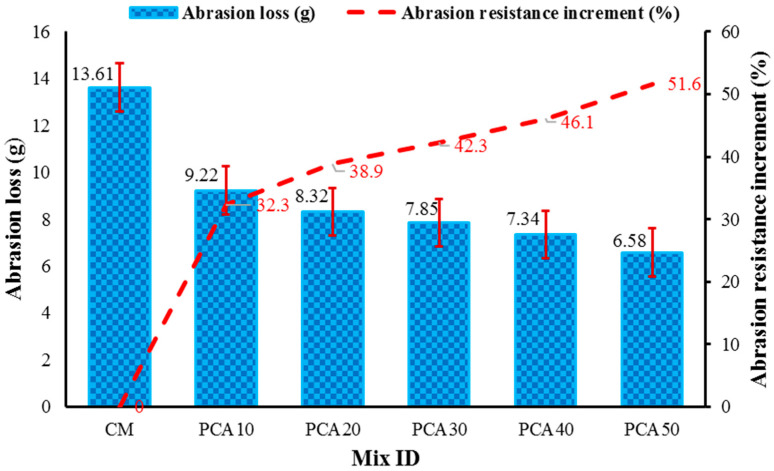
Effect of PCA on abrasion loss of concrete.

Abrasion resistance measures the ability of the concrete surface to counter wearing forces. Concrete abrasion allows the concrete to degrade over time, reducing its toughness by rendering it vulnerable to weathering. The poor resistance of concrete to abrasion makes it more vulnerable to deterioration, which is a common phenomenon. When a concrete structure is subjected to floating ice, a rapid movement of hard and pointed objects, friction, grinding behavior, or other factors, the surface of concrete slowly deteriorates thereby influencing the durability. This test was conducted as per the ASTM C-131 [57] standard. Figure 10 shows the abrasion loss values of RPAC with different percentages of PCA by volume of NCA. The results report that plastic concrete has a considerably high abrasion resistance as compared to the control mix. It can be observed that weight loss reduces with increasing content of PCA. This decremented trend in weight loss with the increasing content of plastic aggregate can be ascribed to the high toughness and greater abrasion resistance of PCA with respect to NCA [7,32,38]. The abrasion resistance of RPAC increased by 32.3%, 38.9%, 42.3%, 46.1%, and 51.6% at 10%, 20%, 30%, 40%, and 50% substitution of NCA, respectively.

#### 3.4.2. Alternate Wetting and Drying

Alternative wetting and drying (W–D) is a durability test conducted to determine the ability of concrete to counter weathering when exposed to W–D conditions such as sea tidal waves. As a result of the alternative W–D of concrete, the stresses are induced in the concrete structure, which cause crack formation. Due to this phenomenon, the reinforcement is subjected to environmental factors such as moisture, air, etc., therefore leading to a decline in the durability characteristics of a structure. In this research work, the 28 days cured concrete specimens of concrete specimens of 100 mm in size were subjected to 25 and 50 alternate W–D cycles. The duration of one complete cycle (two days) means exposing the specimens to wetness for one day and then allowing them to dry for the next day.

**Figure 11:**
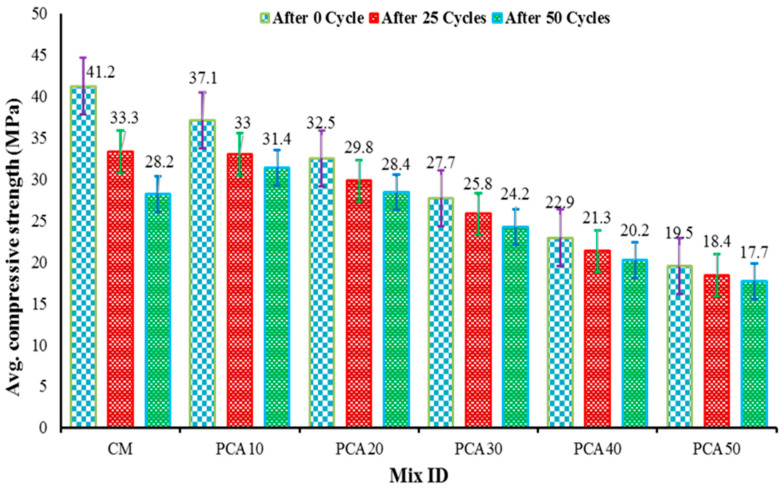
Effect of PCA on alternate wetting and drying of concrete.

Table 6 shows the alternate W–D test results of RPAC after 25 and 50 cycles, along with the average loss in CS. Additionally, Figure 11 shows the comparative CS of RPAC having 0%, 10%, 20%, 30%, 40%, and 50% PCA after 0, 25, and 50 cycles. The results show that wetting and drying the RPAC alternatively significantly influences the CS; however, the decrease in strength declined with the increasing content of plastic aggregates. In other words, increasing the percentage of PCA in concrete increases the resistance of concrete to the CS degradation after subjecting RPAC to alternate wetting and drying. This improved behavior can be attributed to the non-absorbent nature of plastic aggregate. Table 6 shows that the loss of CS in the control mix is 19.17% and 31.55%, respectively, at 25 and 50 cycles while at mix PCA10, the loss of CS reduces to half after 25 and 50 cycles. Similarly, the loss in CS was further reduced at PCA20, PCA30, and PCA40. Finally, the compressive capacity of RPAC containing 50% PCA decreased by only 5.74% and 9.23% with respect to control mix values of 19.17% and 31.55% for 25 and 50 cycles, respectively.

#### 3.4.3. Sorptivity Coefficient

**Figure 12:**
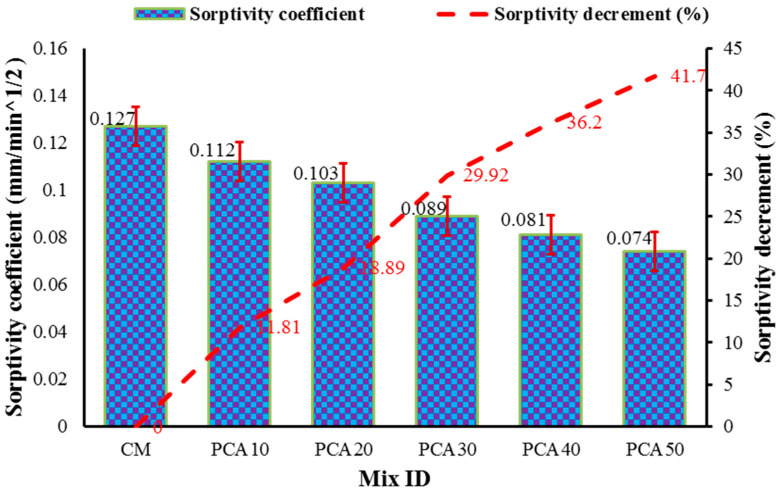
Effect of different percentages of PCA on sorptivity coefficient.

Sorptivity measures the capacity of porous media to absorb water through capillary action. It provides a good measure of the durability of concrete as there are many chemicals that can penetrate the microstructure of concrete from soils and water by capillary action. The sorptivity coefficient is evaluated by determining the rise in the specimen’s weight as a result of the absorption of water through capillary rise with regard to time. The quantity of water absorbed by the samples comprising various proportions of PCA (i.e., 10%, 20%, 30%, 40% and 50%) through capillary rise per unit area is shown in Figure 11. The results show that the sorptivity (mm/min^1/2^) for all the concrete mixes containing plastic aggregates for 28 days of curing has a lesser value than the control mix. This reduction in sorptivity values with reference to the control mix is owing to the zero water absorption capacity PCA [13,32]. With reference to the control mix, the percentage reduction in sorptivity coefficient values of RPAC containing 10%, 20%, 30%, 40% and 50% PCA was 11.81%, 18.89%, 29.92%, 36.2% and 41.7%, respectively, and is shown in Figure 12. Zeeshan et al. [32] found that the incorporation of E-waste plastic aggregate in concrete significantly reduced the sorptivity coefficient value, and observed that at 10%, 15%, and 20% replacement level of PCA with NCA, the sorptivity values decreased by 12.2%, 14.5% and 29%, respectively. Hence, it can be concluded that with the increase in the content of PCA in concrete, the sorptivity coefficient values significantly reduced.

#### 3.4.4. Ultrasonic Pulse Velocity (UPV)

**Figure 13:**
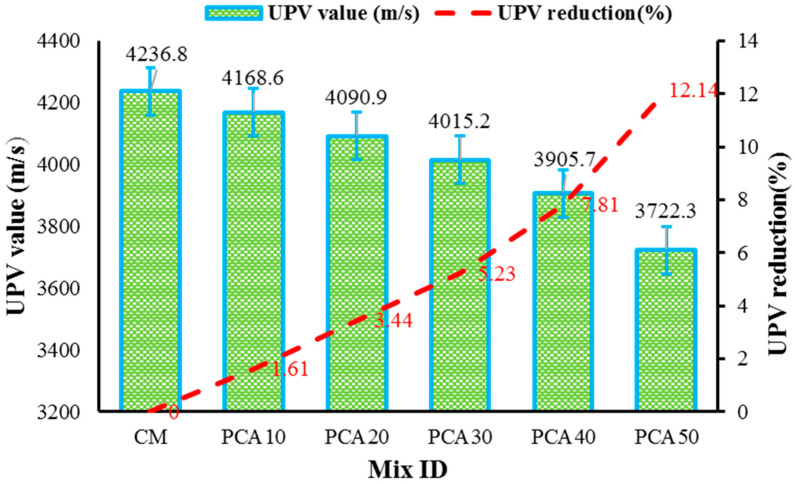
Influence of PCA on UPV value of concrete.

The UPV test was performed to analyze the uniformity, consistency, and quality of the concrete. It also evaluates the imperfections and compactness, i.e., cracks and voids inside a concrete sample. The UPV value has a direct relationship with concrete density, i.e., the denser the concrete, the higher will be its UPV value, and vice versa. The concrete specimen is considered to be of good quality if its UPV value lies in the range of 3.66–4.57 km/s (i.e., 3660–4575 m/s) [58]. Figure 13 shows the UPV values of RPAC containing different percentages of PCA. The results show that with the increase in the percentage of PCA in concrete, the UPV values of RPAC decreased. With reference to the control mix, the UPV values of RPAC containing 10%, 20%, 30%, 40%, and 50% PCA decreased by 1.61%, 3.44%, 5.23%, 7.81%, and 12.14%, respectively. The presence of greater air void content in concrete containing PCA was considered to be the reason for UPV reduction. Therefore, a reduction in the propagation of pulse waves by acoustic impedance occurs. In addition, the UPV value also depends on the elastic properties and volumetric concentration of the various concrete constituents, hence reducing the UPV value when PCA is utilized instead of natural aggregates [32,51]. In the present study, the results reveal that the UPV values for all the specimens lie in the range from 3669 to 4575 m/s; therefore, it is concluded that up to 50% substitution of PCA can be utilized without significantly affecting the quality of concrete.

## 4. Conclusions

This study examined the effect of PCA on the fresh and hardened properties of concrete, including workability, fresh and dry density, CS, STS and FS. Furthermore, durability properties of RPAC such as abrasion resistance, alternate wetting and drying, sorptivity coefficient, and UPV were also explored. In the current study, PCA was used to replace NCA at replacement levels of 10%, 20%, 30%, 40% and 50%. Based on the findings, the following conclusions can be drawn:

1. The incorporation of PCA in concrete significantly increased the workability (16.4–141% for 10% to 50% replacement ratios) due to the zero-water absorption of plastic aggregate. Moreover, the incorporation of PCA in concrete decreased the fresh and dry density of concrete composites for a maximum of 13.6% and 18.2%, respectively, reported at the 50% replacement level of NCA with PCA.
2. The CS, STS and FS of RPAC were considerably reduced by increasing the percentage of PCA. The maximum percentage reduction in CS, STS and FS was achieved as 52.7%, 47.5% and 39.4%, respectively, for 50% replacement.
3. The appreciable reduction (41.7%) in sorptivity value was observed for the maximum replacement of PCA considered in the study. The UPV values also remained within the required range of quality concrete after incorporating the full replacement of PCA. This suggests enhanced durability against the penetration of chemicals into the concrete.
4. The incorporation of PCA in concrete improved the concrete abrasion resistance. Abrasion resistance increased in the range of 32–51% for different substitution ratios. This can be ascribed to the higher abrasion resistance and toughness of plastic aggregate with respect to natural aggregate. Moreover, loss in CS was also reduced after exposure to alternate wetting and drying cycles with respect to the control mix.
5. The manufactured E-waste plastic aggregate can be used to substitute NCA in concrete by volume (10–50%); however, 30% substitution is recommended because up to this level, the characteristic strength of M25 concrete was obtained. Furthermore, the substitution level of 40% and 50% is recommended to be utilized in nonstructural lightweight elements. Additionally, the use of plastic aggregate is recommended to be used in marine conditions owing to its non-absorption capacity, which resists the ingress of hazardous chemicals such as chloride and sulphate, etc. The conventional steel and fiber-reinforced polymer (FRP) rebars in PCA-incorporated concrete are expected to perform better under an alkaline environment; however, more insights regarding the durability of FRPs in PCA-incorporated concrete shall be investigated first from the relevant literature [59,60]. In addition, machine learning techniques are widely used for investigating material properties [61,62,63,64,65,66,67] and general engineering problems [68,69]. Therefore, for the PCA-incorporated concrete, a machine learning regression model can be developed to accurately forecast the strength and durability characteristics for variable input parameters. Future studies need to explore other properties of RPAC, alone or in combination with reinforcing agents such as nanomaterials or fibers, to investigate the mechanical and durability properties, including salt scaling, carbonation, freeze–thaw cycles, concrete to steel bond properties, fire resistance of RPAC, etc.

## Figures and Tables

**Table 1:** General properties of OPC used.

Chemical Composition (Oxides)	Content (% Weight)	Physical Properties	Results
CaO	63.58	Specific gravity	3.14(g·cm^−3^/g·cm^−3^)
SiO_2_	20.4	Specific surface area	321 (m^2^/kg)
Al_2_O_3_	5.10	Consistency	29.15%
Fe_x_O_y_	4.10	Initial setting time	185 min
SO_3_	2.74	Final setting time	241 min
MgO	2.56	Fineness modulus	93.30%
K_2_O	0.88	Compressive strength (28 days)	46.56 (MPa)
Na_2_O	0.23	Soundness	0.103%
Loss on ignition (LOI)	0.41	-	-

**Table 2:** General properties of aggregates.

Property	NCA	PCA	Sand
Max. nominal size (mm)	20	20	4.72
Min. nominal size (mm)	4.75	4.75	0.074
SSD water Absorption (%)	1.08	0	0.5
Specific gravity	2.71	1.21	2.78
Color	Dark	Black brown	Dark
Shape	Angular	Angular	_
Aggregate crushing value	27.42	1.3	NIL
Aggregate impact value (%)	25.43	8.108	NIL
Fineness modulus	NIL	NIL	2.27
Bulk density (g/cm^3^)	1.51	0.49	1.60

**Table 3:** Concrete mix proportions in kg/m^3^.

Mix ID	W/C	Cement	Water	Fine Aggregate	Coarse Aggregate	PCA
CM	0.49	367.34	180	789.14	1133.32	0
PCA10	0.49	367.34	180	789.14	1019.99	52.40
PCA20	0.49	367.34	180	789.14	906.66	104.80
PCA30	0.49	367.34	180	789.14	793.32	157.20
PCA40	0.49	367.34	180	789.14	679.99	209.60
PCA50	0.49	367.34	180	789.14	566.66	262.00

**Table 4:** Testing details.

Test Type	Standard Used	Mix ID					
		0%	10%	20%	30%	40%	50%
Slump test	ASTM C143/C143M-20	3	3	3	3	3	3
Fresh density	ASTM C138/C138M	3	3	3	3	3	3
Dry density	BS EN12390-7	3	3	3	3	3	3
Compressive strength	ASTM C39/C39M	3	3	3	3	3	3
Split tensile strength	ASTM C496/C496M-17	3	3	3	3	3	3
Flexural strength	ASTM C78/C78M-18	3	3	3	3	3	3
Abrasion resistance	ASTM C131/C131-20	3	3	3	3	3	3
Sorptivity coefficient	ASTM C1585-13	3	3	3	3	3	3
UPV	ASTM C597-16	3	3	3	3	3	3
Alternate wetting and drying	_	6	6	6	6	6	6

**Table 5:** Equipment details for each test.

Testing Type	Equipment’s Name	Manufacturer’s Country
Workability	Slump Cone	Turkish Exporter, Istanbol, Turkey
Fresh/dry density	Analytical balance	Pakistan
Compressive strength	Universal Testing Machine (UTM)	Japan
Split tensile strength	Universal Testing machine (UTM)	Japan
Flexural strength	Flexural testing machine	Japan
Sorptivity co-efficient	Cylinder (200 mm thickness & 100 mm dia)	Pakistan
Abrasion resistance	Los Angeles (LA) apparatus	Turkey
Ultrasonic pulse rate	PUNDIT/UPV Apparatus	Japan
Alternating wetting and drying (W–D)	100 mm in side cubes tested using UTM	Pakistan

**Table 6:** Variation in CS after W–D cycles.

Mix ID	After 25 Cycles (MPa)	After 50 Cycles (MPa)	Loss of CS (%)	
			After 25 Cycles	After 50 Cycles
CM	33.3	28.2	19.2	31.6
PCA10	33	31.4	11.1	15.4
PCA20	29.8	28.4	8.31	12.6
PCA30	25.81	24.2	6.86	12.6
PCA40	21.35	20.2	6.73	11.8
PCA50	18.38	17.7	5.74	9.23

## Data Availability

Not applicable.
